# Cytokine Profile in Early Infection by *Leptospira interrogans* in A/J Mice

**DOI:** 10.1155/2019/1892508

**Published:** 2019-10-07

**Authors:** Lorena Bavia, Íris A. de Castro, Mariane Tami Amano, Ana Maria Gonçalves da Silva, Silvio Arruda Vasconcellos, Lourdes Isaac

**Affiliations:** ^1^Department of Immunology, Institute of Biomedical Sciences, University of São Paulo, São Paulo Zip code 05508-900, Brazil; ^2^Institute of Tropical Medicine, University of São Paulo, São Paulo Zip code 05403-000, Brazil; ^3^Department of Preventive Veterinary Medicine and Animal Health, Faculty of Veterinary Medicine and Animal Science, São Paulo Zip code 05508-270, Brazil

## Abstract

Leptospirosis is considered a neglected disease with an estimated more than one million cases every year. Since rodents are at the same time the main reservoir and generally asymptomatic to *Leptospira* infection, understanding why some animal species are resistant and others are susceptible to this infection would shed some light in how to control this important zoonosis. The innate immune response against *Leptospira* is mainly dependent on phagocytosis and activation of the Complement System. In this context, cytokines may drive the early control of infection and the adaptive response. Since the Complement System is important to eliminate leptospires *in vivo*, we investigated if Complement C5 in A/J mice would modulate the cytokine production during infection by *Leptospira interrogans* serovar Kennewicki type Pomona Fromm (LPF). Thus, our aim was to investigate the systemic levels of pro- and anti-inflammatory cytokines during *Leptospira* infection in the blood, liver, lung, and kidney on the third and sixth days of infection in A/J C5^+/+^ and A/J C5^−/−^ mice. Blood levels of TNF-*α*, IL-6, IFN-*γ*, and MCP-1 reached a peak on the third day. Although both mouse strains developed splenomegaly, similar histopathological alterations in the liver and the lung, levels of pro- and anti-inflammatory cytokines were different. A/J C5^+/+^ mice had higher levels of liver IL-10, IL-1*β*, IL-12p40, and IL-12p70 and kidney IL-1*β*, IL-12p40, and IL-12p70 on the sixth day of infection when compared to A/J C5^−/−^ mice. Our results showed that in A/J genetic background, the Complement component C5 modulates a cytokine profile in the liver and kidney of infected mice, which may play a role in the control of disease progression.

## 1. Introduction

Leptospirosis is considered a neglected disease with more than one million cases every year causing approximately 60,000 deaths [1]. This infection is caused by pathogenic *Leptospira*, generally transmitted through abraded or damaged skin or mucosa when in contact with water or soil contaminated with rodents' urine. This disease is an important health issue mainly in developing countries with tropical and subtropical temperatures and with inadequate waste and sewage management systems [2–4]. Leptospirosis is also a risk factor for certain professions such as veterinary and agriculture workers and for practitioners of aquatic sports. Infected individuals can be asymptomatic or they may initially develop fever, myalgia, headache, and jaundice (acute phase) and progress to liver and renal failure and lung hemorrhage (chronic phase) [5]. In the first week, *Leptospira* can be detected in the blood and in the next weeks in the liver, kidney, and lung [6]. Production of specific antibodies is observed only 5-6 days postinfection [7, 8].

In the first days of infection, the innate immune response against *Leptospira* is mainly dependent on phagocytosis and activation of the Complement System [9]. Several important biological functions are generated in consequence of Complement activation such as the production of anaphylatoxins, chemotactic factors, opsonizing fragments, the activation of B lymphocytes stimulating antibody production, and the formation of the membrane attack complex (MAC) leading to microorganism lysis. Complement therefore plays an important role in the overall control of pathogen dissemination and persistence, reviewed in [10].

Complement can be activated by the Classic, Alternative, or Lectin Pathways, and all three converge to a common terminal activation pathway which may lead to MAC-dependent pathogen lysis [11]. The terminal pathway depends on the formation of C5 convertase enzymes which cleave the component C5 in two fragments. C5a, the smaller fragment, is an important anaphylatoxin involved in mast cell and basophile degranulation which releases histamine and other inflammatory mediators like prostaglandins and leukotrienes [12]. C5a is also a well-known chemoattractant factor for neutrophils, monocytes, and eosinophils during acute inflammation. C5b, the largest fragment, is the first to participate in MAC (C5b6789_n_) formation [13, 14]. Besides contributing to control systemic or local infection, the inflammatory properties observed during C5 activation and the participation of receptors such as C5aR1 may be responsible for local tissue damage [15]. The ability to induce cellular lysis and the synergistic interactions with other immune mechanisms highlight the importance of C5 for the immune response.

Nonpathogenic leptospires are rapidly lysed *in vitro* after Complement activation while pathogenic species are more resistant due to several evasion mechanisms that confer protection when the Alternative, Lectin, or Classical pathways are activated [10, 16]. Pathogenic *Leptospira* species can bind to host regulatory proteins, such as Factor H (blocking the alternative pathway), C4b binding protein (avoiding the classical and lectin pathways), and vitronectin (blocking MAC formation) [17–20]. They also secrete proteases that cleave Complement proteins and consequently inhibit all three activation pathways [21] and the MAC formation [22].

Only two studies have investigated the importance of Complement proteins during *in vivo* infection using a murine model. Ferrer et al. [23] observed that in the absence of the Complement regulatory protein Decay-Accelerating Factor (DAF), mice are more prone to develop kidney fibrosis after 90 days postinfection. de Castro et al. [24] explored the importance of C5 in *L. interrogans* serovar Kennewicki strain Fromm (LPF) infection using C5-deficient mice and wild-type mice from the same genetic background (C57BL/6) in the first week of infection. We then observed that C5 is important for controlling the leptospiral load in the liver in the first 3 days of infection.

Although cytokines released during the innate response may drive the early control of infection and the adaptive response, only a few studies investigated the cytokine profile in *Leptospira* infection during immune innate response [25, 26]. In addition, the generation of C5a fragments, and the signaling of its receptors (C5aR1 and C5aR2), may be related to modulating pro- and anti-inflammatory cytokine production [27, 28]. Likewise, the mouse genetic background may also interpose in the inflammatory response. For example, C57BL/6 mice are prone to Th1 response while A/J to Th2 response [29, 30], depending on the infection model. Our previous study showed the liver as the main organ affected by early LPF infection, especially in C57BL/6 C5-deficient mice. However, no difference was found in liver and blood cytokines [24].

In the present study, we employed a different mouse strain (A/J; C5 deficient; C5^−/−^) and evaluated the systemic levels of pro- and anti-inflammatory cytokines during *Leptospira* infection in the blood, liver, lung, and kidney on the third and sixth days after infection. Moreover, to explore the importance of C5 in a different genetic background, we also employed its congenic strain A/J, C5-sufficient (A/J; C5^+/+^) mice generated in our laboratory [31]. Thus, our aim is to investigate the differences in immune responses of A/J C5^+/+^ and A/J C5^−/−^ mice at an early stage of *Leptospira* infection.

## 2. Materials and Methods

### 2.1. *Leptospira* Cultures

Pathogenic LPF cultures were obtained from the Laboratory of Bacterial Zoonosis of Faculty of Veterinary Medicine and Animal Science of the University of São Paulo. Leptospires were kept under aerobic conditions at 29°C for 5-7 days in an Ellinghausen, McCullough, Johnson, and Harris culture medium (EMJH) supplemented with 10% inactivated rabbit serum, L-asparagine (0.015%), sodium pyruvate (0.001%), calcium chloride (0.001%), magnesium chloride (0.001%), peptone (0.03%), and meat extract (0.02%). As a model of virulence, we have used LPF in our studies [24] because infected hamsters become acutely ill and present symptoms such as jaundice, uveitis, light sensitivity, prostration, and loss of appetite when infected with this pathogen. They usually die on day 5 postinfection.

### 2.2. Mice Infection with LPF

We used A/J C5-deficient mouse strain, A/J C5^−/−^ [32], and a congenic A/J mouse strain (A/J C5^+/+^) mice generated in our laboratory with normal C5 levels in the serum [31]. All mice were 4-5 weeks old males obtained from the Animal Facility of the Department of Immunology, Institute of Biomedical Sciences from the University of São Paulo. Mice were i/p infected with 1.5 × 10^8^ LPF in phosphate-buffered saline pH 7.4 (PBS) and euthanized at the third or sixth days postinfection (dpi). Control groups were inoculated only with sterile PBS and euthanized six days later. Hamster is considered a susceptible experimental animal and was used to confirm the virulence by infecting them with LPF culture. This control animal was euthanized when it presented jaundice and reduced mobility. Animals were anaesthetized with ketamine and xylazine (100 mg/kg and 10 mg/kg, respectively) before manipulating. This work was carried out as approved by the Ethics Committee on Animal Experimentation (Certificate 061/10/CEEA). The number of animals used in each experiment is indicated in each figure legend.

### 2.3. Histopathological and Immunochemical Analyses

Liver, lung, and kidney samples were fixed in formalin solution (3.7% formaldehyde in PBS pH 7.4). Microscopy slides were prepared with 5 *μ*m tissue sections stained with hematoxylin-eosin (HE). The histopathological alterations in the liver, lung, and kidney were quantified, as described by de Castro et al. [24]. To quantify the histopathological alterations in the liver, we considered the following criteria: (a) sinusoidal hypercellularity and presence of leucocyte infiltration, (b) presence of mitotic cells, (c) hepatocyte destrabecullation, and (d) cell necrosis. Liver sections with only one of the above criteria were classified with a score of 1; with two of above criteria, a score of 2; with three of the above criteria, a score of 3; and with all the above criteria, a score of 4. To quantify the histopathological alterations in the lung, we considered the following criteria: (a) presence of nodular interstitial pneumonitis (IP) was classified with a score of 1; (b) presence of diffuse IP was classified with a score of 2. Due to the lack of renal lesions, there was no classification of this organ in this work.

In addition, liver immunohistochemical analyses were performed to assess the presence of leptospiral antigens using rabbit antileptospire serum, according to de Castro et al. [24]. For a semiquantitative evaluation of bacterial load, the average area (%) of leptospiral antigens in hepatic tissue was measured using an image analyzer program in seven randomly selected fields at 200x magnification.

### 2.4. Biochemical Assays

Hepatic function integrity was indirectly evaluated by measuring alanine transaminase (ALT) and aspartate transaminase (AST) serum concentrations, while kidney function was evaluated by urea and uric acid (Bioclin Quibasa, Belo Horizonte, MG, Brazil) serum levels as described by Bavia et al. [31].

### 2.5. Blood Leukocyte Counting

Fresh blood samples were obtained from orbital venous plexus with heparinized glass capillary tubes from anesthetized mice. Samples were diluted in Türk solution (4.76 mM acetic acid, 6.25 *μ*M methylene blue), and the total number of peripheral blood leukocytes was counted using a Neubauer chamber.

### 2.6. Cytokine Measurements

Levels of TNF-*α*, IL-1*β*, IL-6, IL-10, IL-12p40, and IL-12p70 in the organs were determined by ELISA, as described in Bavia et al. [33]. Serum levels of TNF-*α*, IFN-*γ*, IL-6, IL-12p70, MCP-1, and IL-10 were determined using the Inflammation CBA kit (BD Bioscience), according to the manufacturer's instructions. Data was acquired using the FACSCanto II flow cytometer (BD Bioscience, Franklin Lakes, New Jersey, United States of America) and data analysis by FCAP Array™ v3.0.1 Software (BD Bioscience, Franklin Lakes, New Jersey, United States of America).

### 2.7. Statistical Analysis

Normal distributions of the continuous variables were tested by the Kolmogorov-Smirnov method. Continuous data were expressed as mean and standard error. The results were submitted to ANOVA two-way with Bonferroni posttest. All analyses considered a significance level of at least 95% (*p* < 0.05).

## 3. Results

### 3.1. Physiological Profile in A/J Mouse Strain Infected by LPF

Considering that *Leptospira* is observed in the circulation at the onset of infection [5], we followed the development of splenomegaly and evaluated the amount of circulating leukocytes in the LPF-infected mice (Table 1). Our results showed an increase in the ratio of spleen and body weight on the third and the sixth days of infection when compared to the PBS control group in both mouse strains. On the other hand, the number of peripheral leukocytes in the A/J C5^+/+^ mice decreased on the third day postinfection and increased significantly from the third to the sixth days after infection.

Hepatic damage was indirectly evaluated by measuring the serum levels of alanine transaminase (ALT) and aspartate transaminase (AST), while kidney function was evaluated by urea and uric acid serum concentrations. The A/J C5^+/+^ mouse strain presented significantly increased ALT and AST levels on the third day of infection when compared to the PBS control, followed by a significant reduction of these enzymes on the sixth day versus the third day of infection. In contrast, serum urea concentration decreased significantly in A/J C5^+/+^ mice on the third day followed by partial recovery of the initial concentrations on the sixth day of infection. In the A/J C5^−/−^ mice, urea was significantly reduced on both the third and the sixth days when compared to the PBS control. No differences in the serum uric acid concentration were observed in both A/J mouse strains.

### 3.2. Histopathological Analysis in LPF-Infected Mice

**Figure 1:**
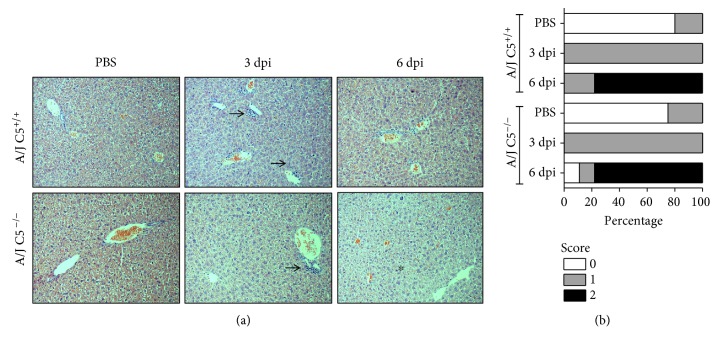
Liver histopathological analysis in LPF-infected A/J mice. Mice were inoculated i/p with 1.5 × 10^8^ LPF or only PBS and then euthanized on the third or the sixth days postinfection (*n* ≥ 4). (a) Liver sections (3–5 *μ*m) were stained with HE and evaluated at 200x magnification. Arrows indicate leukocyte infiltration in the portal space and in the hepatic sinusoids. Asterisk indicates mitotic cells. (b) Total scores of hepatic lesions. A/J^−/−^: C5-deficient mice, PBS (*n* = 5), 3 dpi (*n* = 12), 6 dpi (*n* = 9); A/J C5^+/+^: congenic C5-sufficient mice PBS (*n* = 4), 3 dpi (*n* = 10), 6 dpi (*n* = 9). dpi: days postinfection.

**Figure 2:**
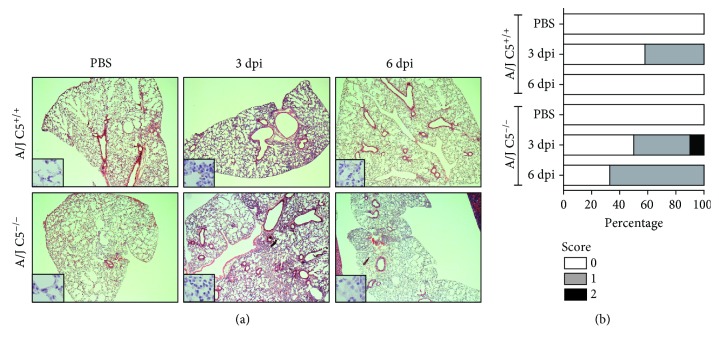
Lung histopathological analysis in LPF-infected A/J mice. Mice were inoculated i/p with 1.5 × 10^8^ LPF or only PBS and then euthanized on the third or the sixth days postinfection (*n* ≥ 4). (a) Lung sections (3–5 *μ*m) were stained with HE and evaluated at 200x magnification. The inserts present the immunohistochemical analysis of the lung from LPF-infected mice evaluated at 400x magnification. (b) Total scores of lung lesions. A/J^−/−^: C5-deficient mice, PBS (*n* = 5), 3 dpi (*n* = 12), 6 dpi (*n* = 9); A/J C5^+/+^: congenic C5-sufficient mice PBS (*n* = 4), 3 dpi (*n* = 10), 6 dpi (*n* = 9).dpi: days postinfection.

**Figure 3:**
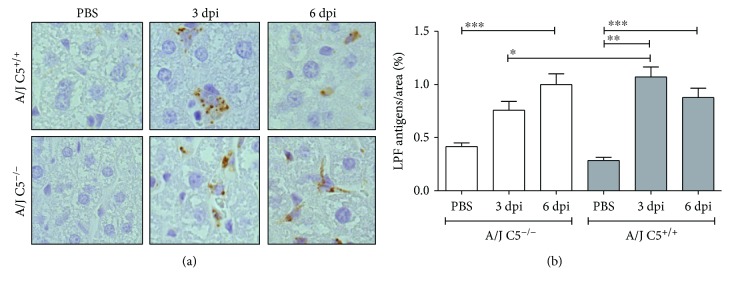
Presence of LPF antigens in the liver of LPF-infected A/J mice. (a) Liver sections from A/J mice were incubated with rabbit antileptospire immune serum. (b) Semiquantitative measurements of LPF antigens in the liver of infected A/J mice were performed in seven random fields of each section. Significant differences are indicated as ^∗^ when *p* < 0.05, ^∗∗^*p* < 0.01, and ^∗∗∗^*p* < 0.001. A/J^−/−^: C5-deficient mice, PBS (*n* = 5), 3 dpi (*n* = 12), 6 dpi (*n* = 9); A/J C5^+/+^: congenic C5-sufficient mice PBS (*n* = 4), 3 dpi (*n* = 10), 6 dpi (*n* = 9). dpi: days postinfection.

Histopathological changes were observed in the liver (Figure 1) and the lung (Figure 2) but not in the kidney on the third and sixth days of infection in all animals during the experiment (Supplementary [Supplementary-material supplementary-material-1]). Leukocyte infiltration was observed around the portal spaces and within the hepatic sinusoids and increased the number of Küpffer cells in the sinusoids in all groups infected with LPF (Figure 1(a)). On the sixth day of infection, there was a reduction in the number and size of leukocyte infiltration and the appearance of mitotic cells in the liver of both mouse strains. The degree of hepatic injury was similar between A/J C5^+/+^ and A/J C5^−/−^ mice, and few variations were observed between the third and the sixth days of infection (Figure 1(b)). The presence of LPF antigens in the liver of infected mice was determined by immunohistochemical analysis (Figure 3) using rabbit antileptospire immune serum. The percentage of labeled areas was significantly higher in A/J C5^+/+^ when compared to A/J C5^−/−^. However, this percentage was very low (~1.1% and 0.76%, respectively) in both mouse strains.

The histopathological analysis of the lungs showed regions with increased thickness of the alveolar septum (interstitial pneumonitis). Lungs of A/J C5^+/+^ mice had isolated foci of these lesions only on the third day of infection. A/J C5^−/−^ had isolated foci of interstitial pneumonitis on the third and the sixth days of infection. In addition, generalized interstitial pneumonitis was observed in the lung samples of some A/J C5^−/−^ animals on the sixth day (Figures 2(a)–2(b)). Practically, no LPF antigens were observed in the lungs of infected mice (see the insert in Figure 2(a)).

As shown in Table 1, both mouse strains employed in our study developed splenomegaly through LPF infection. We did not find renal changes that could have been caused by the infection since this organ morphology was similar in infected animals and the noninfected controls (Supplementary [Supplementary-material supplementary-material-1]).

Together, these results suggest that in the early days of LPF infection, the liver and lung were affected in both mouse groups. Total lesion scores observed in the lungs of A/J C5^−/−^ mice were higher at the sixth day of LPF infection when compared to congenic A/J C5^+/+^ mice.

### 3.3. Cytokine Profile in A/J Mouse Strain Infected by LPF

#### 3.3.1. Serum Cytokines

Serum concentrations of TNF-*α* were significantly higher on the third and sixth days of infection when compared to the PBS control, for both A/J C5^+/+^ and A/J C5^−/−^ mice. Serum concentrations of IFN-*γ* and IL-6 followed the same profile observed for TNF-*α* concentration, and MCP-1 levels were detected only on the third day after infection. Serum levels of IL-10 and IL-12p70 were undetectable (Table 2).

#### 3.3.2. Liver, Lung, and Kidney Cytokines

**Figure 4:**
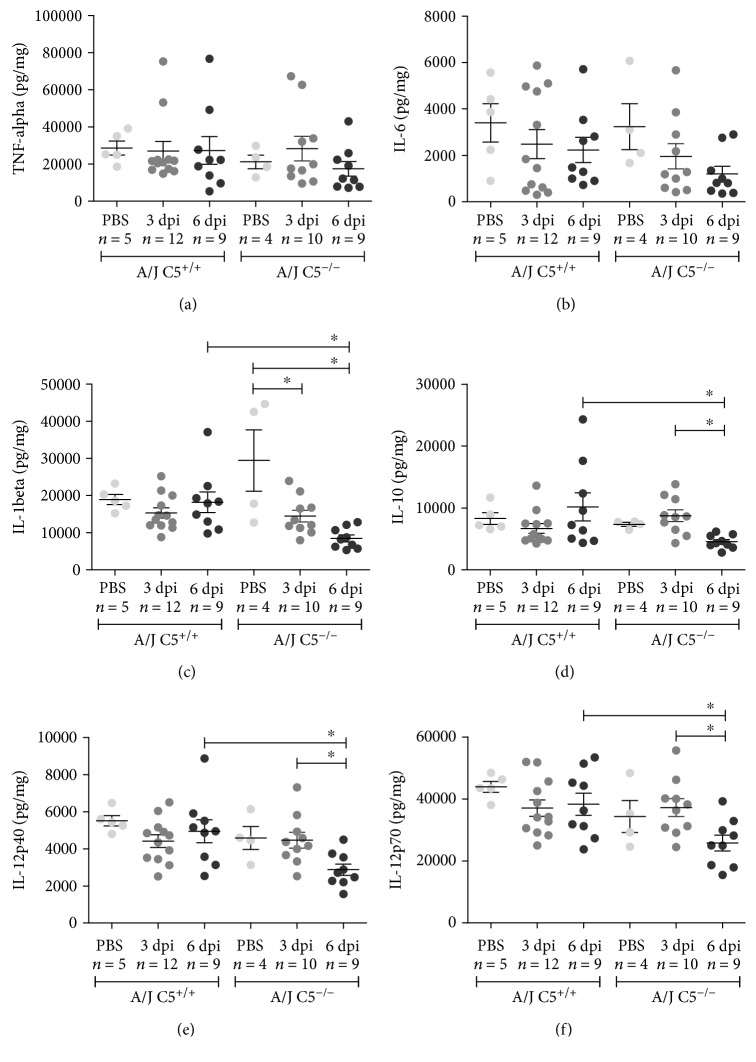
Liver concentrations of pro- and anti-inflammatory cytokines during early infection by LPF. A/J C5^+/+^ and A/J C5^−/−^ mice were inoculated i/p with 1.5 × 10^8^ LPF or PBS and then euthanized on the third or the sixth days. The concentrations of several cytokines were analyzed in the liver extracts by ELISA, using the same protein concentration in each sample. Significant differences are indicated as ^∗^ when *p* < 0.05. dpi: days postinfection.

No differences were observed for hepatic levels of TNF-*α* and IL-6 cytokines (Figures 4(a) and 4(b)). The hepatic levels of IL-1*β*, IL-10, IL-12p40, and IL-12p70 were significantly reduced on the sixth day of infection in A/J C5^−/−^ mice when compared to A/J C5^+/+^ (Figures 4(c)–4(f)). Also, we observed that among the A/J C5^−/−^ mice, the concentrations of these cytokines were also lower in relation to the third day of infection (Figures 4(c) and 4(d)).

**Figure 5:**
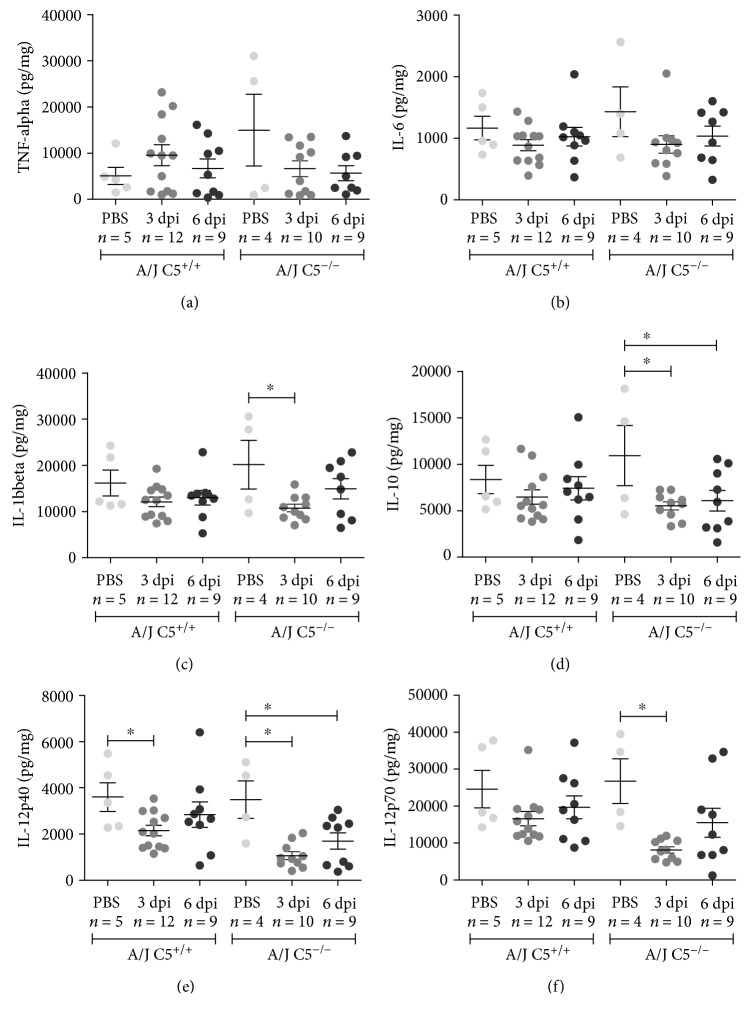
Lung concentrations of pro- and anti-inflammatory cytokines during early infection by LPF. A/J C5^+/+^ and A/J C5^−/−^ mice were inoculated i/p with 1.5 × 10^8^ LPF or PBS and then euthanized on the third or the sixth days. The concentrations of several cytokines were analyzed in the liver extracts by ELISA, using the same protein concentration in each sample. Significant differences are indicated as ^∗^ when *p* < 0.05. dpi: days postinfection.

The lung levels of IL-1*β*, IL-10, IL-12p40, and IL-12p70 were significantly reduced on the third day of infection when compared to the PBS control in A/J C5^−/−^ mice (Figures 5(c)–5(f)). Similar result was observed in the lung level of IL-12p40 in A/J C5^+/+^ mice (Figure 5(e)). Moreover, lung levels of IL-10 and IL-12p40 remained reduced on the sixth day of infection when compared to the PBS control in A/J C5^−/−^ mice (Figures 5(d) and 5(e)). No significant differences were observed for TNF-*α* and IL-6 cytokines (Figures 5(a) and 5(b)) in the lung.

**Figure 6:**
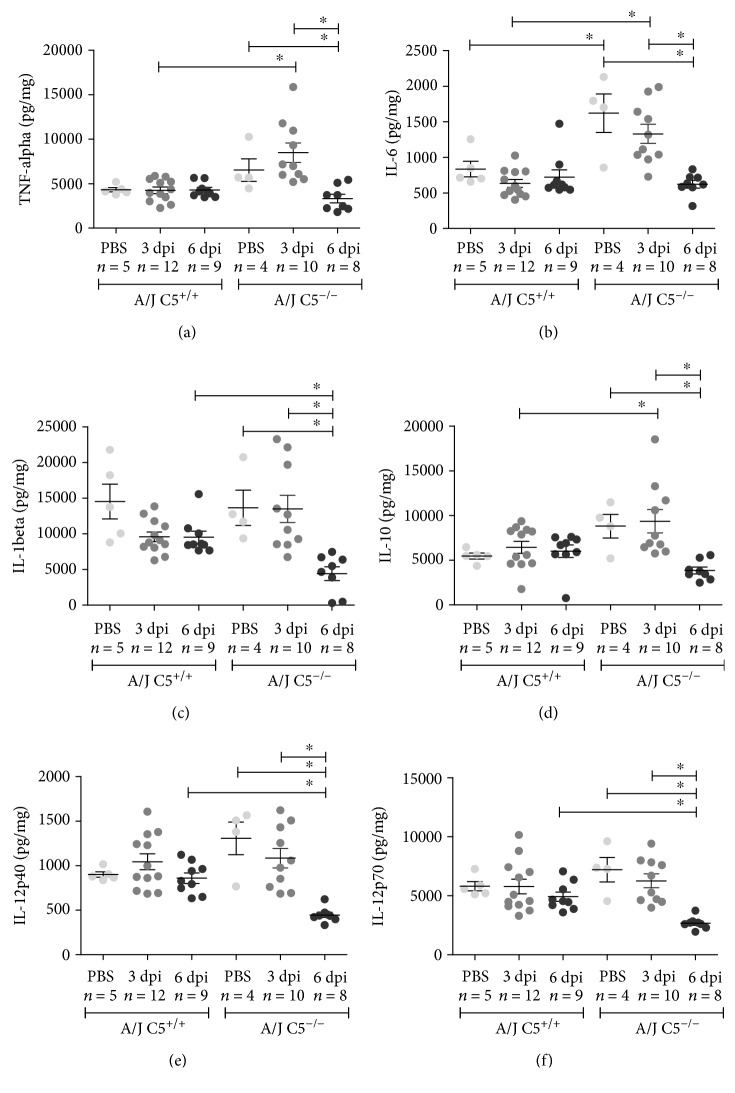
Kidney concentrations of pro- and anti-inflammatory cytokines during early infection by LPF. A/J C5^+/+^ and A/J C5^−/−^ mice were inoculated i/p with 1.5 × 10^8^ LPF or PBS and then euthanized on the third or the sixth days. The concentrations of several cytokines were analyzed in the liver extracts by ELISA, using the same protein concentration in each sample. Significant differences are indicated as ^∗^ when *p* < 0.05. dpi: days postinfection.

Regarding kidney cytokines, significant differences were observed between A/J C5^+/+^ and A/J C5^−/−^ mice. While kidney levels of TNF-*α*, IL-6, and IL-10 were significantly increased on the third day after infection, the levels of IL-1, IL-12p40, and IL-12p70 were significantly reduced on the sixth day after infection in A/J C5^−/−^ mice when compared to A/J C5^+/+^. In addition, in A/J C5^−/−^ mice, levels of all cytokines evaluated were higher in PBS control and third day after infection when compared to sixth day after infection (Figures 6(a)–6(f)).

Taken together, the results show that LPF infection triggered the production of pro- and anti-inflammatory cytokines in all organs evaluated. In addition, except for TNF-*α* and IL-6, the cytokine profile is different in the liver and kidney of A/J C5^+/+^ and A/J C5^−/−^ mouse strains. However, these differences could not be attributed to the leptospiral load in both mouse strains.

**Figure 7:**
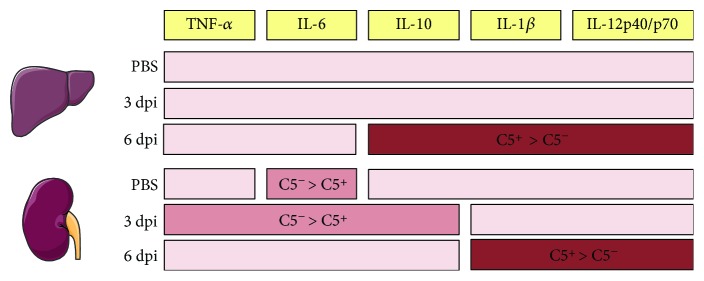
Cytokine levels are dependent on the presence of Complement component C5 during early infection by LPF. Pro- and anti-inflammatory cytokines were evaluated in the liver, lung, and kidney. The levels of IL-10, IL-1*β*, and IL-12 (p40 and p70) were higher in the liver of A/J C5^+/+^ (C5^+^) versus A/J C5^−/−^ (C5^−^) mice on the sixth day of infection. Although the kidneys are the organs affected in the chronic period of LPF infection, the lack of C5 was important for the production of TNF-*α*, IL-6, and IL-10 on the third day of infection. However, the presence of C5 promoted the synthesis of IL-1*β* and IL-12 (p40 and p70). Since no difference was found in lung cytokine levels regarding the presence or lack of C5, this organ was omitted. dpi: days postinfection.

In order to more clearly indicate the influence of C5 in the cytokine profile in our model of LPF infection, all C5-dependent results are presented in Figure 7.

## 4. Discussion

The early stages of immune response in leptospirosis infection are crucial for clinical outcomes. Thus, to understand the complex network of cytokine action during infection remains an area of intense interest. Although the murine model is considered resistant for leptospirosis infection, it allows the study of responses mediated by specific effector immune molecules. In this context, we have demonstrated that the C5 component plays an important role in the modulation of cytokines involved with the early establishment of LPF infection mainly in the kidneys of A/J mice.

In this work and other studies [21, 24, 34], we used LPF because infected hamsters become acutely ill and present symptoms such as jaundice, uveitis, light sensitivity, prostration, and loss of appetite. They usually die on day 5 postinfection. A widely used *L. interrogans* serovar Copenhageni Fiocruz L1-130 has been shown to cause milder symptoms and delayed death when compared to the LPF strain.

In our model of acute infection, no differences in the development of splenomegaly were observed between A/J C5^+/+^ and A/J C5^−/−^ mice. In our previous study, C57BL/6 mice infected with the similar amount of LPF also presented splenomegaly on the third day; however, a regression of spleen size was observed on the sixth day of infection [24]. Corroborating these findings, C57BL/6 mice infected with *L. interrogans* serovar Autumnalis presented a massive splenic enlargement within 3 days postinfection [35]. Moreover, in our study, both mouse strain groups had a transient weight loss on the third day of infection with recovery on the sixth day. Furthermore, our results are in agreement with Rated et al. [6] that demonstrated the course of experimental infection with a sublethal dose of *L. interrogans* serovar Manilae strain L495 as a biphasic illness. The first week of infection begins with a mild acute phase, characterized by septicemia associated with transient weight loss, and subsequent clearance of the bacteria in the blood. The chronic phase starts on the second week and is characterized by establishment of an asymptomatic renal *L. interrogans* colonization and excretion of leptospires in the urine [6].

One of the common procedures to verify the intensity of leptospirosis in the body is through the measurement of serum biochemical parameters that indicate the presence of liver damage, such as ALT and AST transaminases, and renal activity, such as urea and uric acid. The LPF infection caused increased levels of ALT and AST on the third day of infection in A/J C5^+/+^ mice, suggesting that the presence of C5 may facilitate hepatocyte injury recruiting leukocytes to eliminated LPF in the liver of A/J mice, different from that observed for C57BL/6 mice for which no difference was observed [24]. In addition, the levels of urea decreased in both A/J C5^+/+^- and A/J C5^−/−^-infected mice. Since urea is a product of protein breakdown and its synthesis occurs mainly in the liver, the peripheral urea nitrogen concentration reflects protein metabolism; the reduction observed in infected mice may reflect the reduction of food intake and, consequently, weight lost [36].

Complement component C5 has been implicated as an important factor for liver regeneration after injury [37–39]. In our model of LPF infection, both A/J C5^+/+^ and A/J C5^−/−^ mice showed leukocyte infiltration on the third day and mitotic cells on the sixth day of infection. However, both C57BL/6 C5^+/+^ and C57BL/6 C5^−/−^ mice also presented leukocyte infiltration on the third and sixth days of infection. Although C5 is important for the synthesis of growth factors in the injured tissue to stimulate cell proliferation [38], our results also showed the importance of mouse genetic background. On the other hand, C5 also contributes to tissue injury in different experimental models [40–43] due to the ability of C5a to stimulate and attract several cell types to the site of inflammation [44–48]. While A/J C5^+/+^ and A/J C5^−/−^ infected with LPF showed similar liver injury scores, C57BL/6 C5^+/+^ mice showed more liver injury than C57BL/6 C5^−/−^ mice, suggesting the lack of C5 as a protective factor during LPF infection in C57BL/6 genetic background [24]. It is noteworthy that our results with A/J and C57BL/6 mice showed a synergism between C5 and mouse genetic background during LPF infection. It is known that C57BL/6 mice are more prone to proinflammatory immune profile than A/J mice [29, 30]. Lung injury in our model was similar to that observed by da Silva et al. [25]. However, only A/J C5^+/+^ mice presented lung recovery on the sixth day of infection when compared with A/J C5^−/−^, C57BL/6 C5^+/+^, and C57BL/6 C5^−/−^ [24]. Furthermore, we expected a large amount of LPF in the alveolar septa, but this was not confirmed by immunohistochemistry, suggesting that lung injury was not directly caused by leptospires. Finally, we did not observe renal lesions or detect leptospires in the kidney of the A/J mice in our experimental model as observed before with C57BL/6 mice [24]. Thus, our results are inconsistent with the kinetics of leptospiral infection studied by other authors [6] when C57BL/6 mice were infected with bioluminescent *L. interrogans* serovar Manilae strain L495. They demonstrated that kidneys were the main reservoir of leptospires during the chronic phase. One possible explanation is that LPF infection has particular characteristics and clinical consequences that differ from those caused by other pathogenic leptospires more commonly used by other researchers.

The fragment C5a can act as an anaphylatoxin binding to C5aR1 in mononuclear macrophages, granulocytes, mast cells, and platelets that amplify inflammation, and the fragment C5b is essential for the MAC formation [11]. Furthermore, during the infection, the presence of C5 and its receptor C5aR1 may represent a double-edged sword. In the model of cerebral malaria, C57BL/6 mice (C5 sufficient) are susceptible to infection, whereas the A/J mice (C5 deficient) are resistant. To investigate whether this susceptibility would be associated with the presence of C5 or the different mouse genetic background, Patel et al. generated C57BL/6 C5-deficient and A/J C5-sufficient congenic mice and subjected these animals to infection with *Plasmodium berghei* [41]. Interestingly, these authors observed that A/J C5-sufficient mice became susceptible to the development of cerebral malaria, confirming that in the experimental murine model, C5 contributed to the pathogenesis of cerebral malaria independently of genetic background. Likewise, C5 or C5aR-deficient mice were also resistant to developing sepsis in the experimental model of cecal ligation and puncture [49]. On the other hand, while C5-deficient mice displayed higher susceptibility to *Neisseria meningitidis* infection, the lack of C5aR1 contributed to a favourable outcome ameliorating inflammatory cytokine response more rapidly than wild-type mice [50].

While in A/J C5^+/+^ mice we observed a recovery in the number of leukocytes from the third to the sixth day of infection, in A/J C5^−/−^, no difference was observed. This result may be related to two conditions: (1) migration of cells to the organs affected by LPF, such as the liver (leukocyte infiltration in the portal system and sinusoids) and the lung (interstitial pneumonitis), and (2) lower generation of leukocytes in the bone marrow due to the increased concentration of IFN-*γ* and TNF-*α* in the serum of A/J C5^−/−^ mice. It is known that the presence of IFN-*γ* and TNF-*α* at high concentrations is detrimental to the proliferation of hematopoietic cells, reducing their ability to replicate and increasing the rate of apoptosis [51]. An experimental model of anemia also demonstrated the suppressive role of these cytokines on hematopoietic progenitors [52, 53] supporting the hypothesis of a relationship between the decrease in the number of leukocytes on the third day with the increase of IFN-*γ* and TNF-*α* in the serum of both A/J C5^+/+^ and A/J C5^−/−^ mice. Furthermore, in our previous study, both C57BL/6 C5^+/+^ and C57BL/6 C5^−/−^ mice when infected with LPF showed decreased levels of circulating leukocytes on the sixth day of infection concomitantly with an increase in serum IFN-*γ* [24].

Differences in the levels of some cytokines between A/J C5^+/+^ and A/J C5^−/−^ mice were observed in our LPF infection model. A/J C5^+/+^ mice had higher levels of liver IL-10, IL-1*β*, IL-12p40, and IL-12p70 and kidney IL-1*β*, IL-12p40, and IL-12p70 on the sixth day of infection than A/J C5^−/−^. In line with our findings, the lack of C5 [54] or C5aR blocking [55] resulted in lower synthesis of different cytokines, such as IL-6 and IL-12p40, when the T lymphocytes of these animals are stimulated. In addition, the reduction of IL-12 p40 subunit levels may lead to lower production of IL-12p70, since this cytokine is formed by the covalent attachment of p35 and p40 subunits [56, 57]. Furthermore, the signaling by C5a, together with TNF-*α*, acts as priming signal 1 for NLRP3 activation by increasing *IL1B* gene transcription, as reviewed by Arbore and Kemper [58]. Consequently, the high levels of IL-10 in infected A/J C5^+/+^ mice may be associated with the high levels of IL-1*β*, since this cytokine is able to regulate IL-10 synthesis [59]. This concomitant variation in levels of IL-10 and IL-1*β* was observed before in the experimental model of sepsis, in which the lack of C5 resulted in lower levels of both cytokines [60].

In addition, IL-10 mediates immunosuppression while IL-12 enhances cellular immune response. However, our results show that both IL-10 and IL-12 levels are elevated in the liver and kidneys (only IL-12) of A/J C5^+/+^-infected mice. IL-10 and IL-12 synthesis may be triggered by different kinds of stimulus, such as molecules from pathogenic microorganisms or antigens delivered during an inflammatory process. When stimulated by LPS, mouse macrophages produce higher levels of IL-12 than nonstimulated macrophages [61]. Likewise, the production of IL-10 may be dependent of LPS. First, the stimulation by LPS through TLR4 induces the transcription of Type I IFNs (IFNs beta/alpha). Then, autocrine or paracrine signaling via the Type I IFN Receptor (IFNaR) leads to induction of the IL-27. Consequently, IL-27 signaling triggers the synthesis and phosphorylation of STAT-1 and STAT-3, which are important in the final pathway for the synthesis of IL-10 by the cells [62]. Furthermore, different authors have associated the presence of C5a to higher activity of STAT-3 during inflammatory responses and cell proliferation. C5a signaling increases acetylation of STAT-3 gene regions, turning those regions more accessible to the nuclear transcription machinery [37, 63], and also promotes STAT-3 phosphorylation during inflammatory response [37, 64], which could result in IL-10 synthesis as observed in A/J C5^+/+^ mice at the sixth day of infection.

The cytokines' levels during the early infection with *L. interrogans* serovar Copenhageni were previously determined by da Silva et al. [25]. They observed that liver TNF-*α* levels significantly increased in two different mouse strains (C3H/HePas and Balb/c) but not in C3H/HeJ mice at the third day of infection. In our experimental conditions using LPF in A/J (this study) or C57BL/6 [24] mice, no significant differences were observed suggesting that the genetic background and species of pathogenic *Leptospira* influence the expression of this cytokine in the liver. In addition, in agreement with data from da Silva et al. [25], the TNF-*α* levels in the kidney increased significantly in both A/J mouse strains (C5^+/+^ and C5^−/−^) when compared to the PBS control group. Noteworthy, A/J C5^−/−^ mice have higher levels of TNF-*α*, IL-6, and IL-10 in the kidney when compared to A/J C5^+/+^ mice on the third day of infection. This increase in cytokine concentration in the C5^−/−^ mouse strain was also observed in *C. albicans* infection [42] and asthma models [65]. A/J C5^−/−^, C57BL/6 C5^−/−^, or C5aR-deficient mice show higher concentrations of different serum cytokines than C5- and/or C5aR-sufficient mice, including IL-6, when infected with *C. albicans* [42]. Likewise, IL-6 levels increased in bone marrow-derived DCs from A/J mice when compared with C3H/HeJ [65]. Interestingly, A/J and C57BL/6 mice when infected chronically for 28 days with a low (10^3^) or a high (10^6^) infective dose of *L. interrogans* serogroup Icterohaemorrhagiae strain Cop presented similar dose-independent nephritis [66]. Unfortunately, renal cytokines were not evaluated by Santos et al. [66] neither in our previous study [24]. In a similar chronic model of infection by *L. borgpetersenii* serogroup Ballum, cytokines IL-1*β* and IL-10 were upregulated in hamster kidneys compared to OF1 mouse strain after 14 and 21-28 days postinfection. TNF-*α* expression increased in both hamster and mice at 21-28 days postinfection [26].

Thus, understanding why some animal species are resistant and other are susceptible to this infection and how the complex network of cytokines acts during infection would contribute to treat, combat, and prevent this important zoonosis.

## 5. Conclusions

The levels of proinflammatory cytokines may vary according to the genetic background during pathogenic LPF infection. The Complement component C5 modulates some cytokine profile in the liver and kidney of A/J-infected mice during early days of LPF infection.

## Figures and Tables

**Table 1:** Physiological profile of A/J mouse strain infected by LPF.

		A/J C5^+/+^			A/J C5^−/−^	
	PBS (n = 5)	3 dpi (*n* = 12)	6 dpi (*n* = 9)	PBS (*n* = 4)	3 dpi (*n* = 10)	6 dpi (*n* = 9)
Weight gain (%)	12.6 ± 1.7	1.5 ± 0.6^a^	6.5 ± 2.1^a^	8.3 ± 2.1	3.1 ± 0.7	12.4 ± 2.4^bc^
Splenomegaly (%)	0.36 ± 0.04	0.65 ± 0.04^a^	0.64 ± 0.06^a^	0.23 ± 0.05	0.49 ± 0.05^a^	0.63 ± 0.04^a^
Circulating leukocytes (10^6^ cells/ml)	8.9 ± 0.9	5.6 ± 0.9	9.3 ± 0.9^b^	8.2 ± 1.3	6.0 ± 0.8	7.4 ± 0.8
ALT (U/dl)	9.0 ± 1.9	27.4 ± 3.3^a^	17.1 ± 2.4^b^	11.6 ± 2.8	21.5 ± 3.0	11.4 ± 2.1^b^
AST (U/dl)	15.0 ± 0.9	32.2 ± 6.2^a^	15.0 ± 2.0^b^	14.7 ± 2.7	25.0 ± 1.7	17.3 ± 2.6
Urea (mg/dl)	52.7 ± 3.1	41.9 ± 0.9^a^	50.2 ± 2.2^b^	55.2 ± 2.3	46.7 ± 2.06^a^	47.2 ± 1.05^a^
Uric acid (mg/dl)	1.1 ± 0.2	1.4 ± 0.3	0.7 ± 0.5	1.1 ± 0.2	1.4 ± 0.2	0.7 ± 0.1

Notes: the body weight was recorded immediately before the infection and at the end of the experiment. The spleen weight was normalized by the body weight at the end of the experiment. Abbreviations: A/J C5^+/+^ (A/J C5-sufficient mice), A/J C5^−/−^ (A/J C5-deficient mice); dpi: days postinfection. Statistical representation: a *vs.* PBS; b *vs.* 3 dpi; c *vs.* A/J C5^+/+^. All data are presented as mean and standard error.

**Table 2:** Serum cytokine levels in A/J mouse strain infected by LPF.

		A/J C5^+/+^			A/J C5^−/−^	
	PBS (*n* = 5)	3 dpi (*n* = 12)	6 dpi (*n* = 9)	PBS (*n* = 4)	3 dpi (*n* = 10)	6 dpi (*n* = 9)
TNF-*α* (pg/ml)	3.4 ± 0.8	18.1 ± 2.7^a^	6.8 ± 2.2^b^	4.8 ± 0.6	21.8 ± 4.9^a^	5.2 ± 0.8^b^
IFN-*γ* (pg/ml)	un	3.9 ± 0.3	0.5 ± 0.5	0.04 ± 0.04	45.8 ± 34.6	0.16 ± 0.1
IL-6 (pg/ml)	un	2.3 ± 0.9	0.2 ± 0.2	un	4.4 ± 1.2	0.03 ± 0.04
MCP-1 (pg/ml)	un	14.1 ± 4.6	un	un	38.4 ± 19.0	un

The concentrations of cytokines were analyzed in the serum of infected mice by flow cytometry (CBA). Abbreviations: A/J C5^+/+^ (A/J C5-sufficient mice), A/J C5^−/−^ (A/J C5-deficient mice), dpi: days postinfection; un: undetected. Statistical representation: a *vs.* PBS; b *vs.* 3 dpi. All data are presented as mean and standard error.

## Data Availability

The data used to support the findings of this study are included within the article.

## Supplementary Materials

Supplementary Materials Supplementary Figure 1 Kidney histopathological analysis in LPF-infected A/J mice.
