# Supplementary material for: Cytokine Profile in Early Infection by Leptospira interrogans in A/J Mice
Source: J Immunol Res. 2019 Oct 7;2019:1892508. doi: 10.1155/2019/1892508 (PMC6800925; doi:10.1155/2019/1892508)
Supplement: Supplementary Materials — Supplementary Figure 1 Kidney histopathological analysis in LPF-infected A/J mice. [file 1892508.f1.pdf]

Supplementary Materials

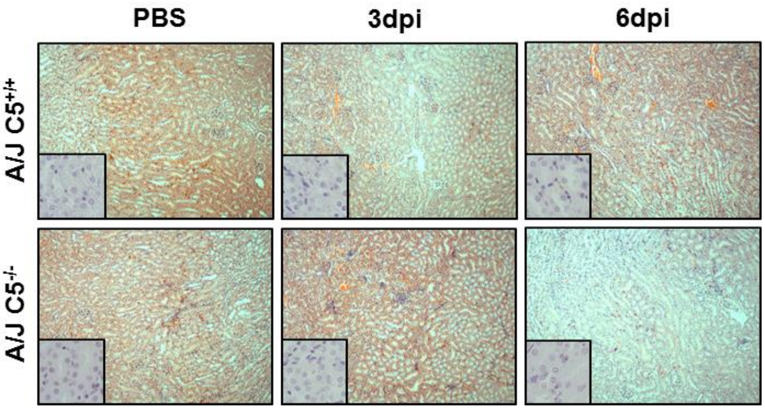

Supplementary figure 1: Kidney histopathological analysis in LPF infected A/J mice. Mice were inoculated i/p with 1.5×10^8 LPF or only PBS and then euthanized on the third or the sixth days post-infection ($n \geq 4$). Kidney sections (3–5 μm) were stained with (HE) and evaluated at 200x magnification. The inserts present the immunohistochemical analysis of kidney from infected mice evaluated at 400x magnification. A/J$^-$: C5 deficient mice; A/J C5$^{+/+}$: congenic C5 sufficient mice.
